# African Horse Sickness: A Review of Current Understanding and Vaccine Development

**DOI:** 10.3390/v11090844

**Published:** 2019-09-11

**Authors:** Susan J Dennis, Ann E Meyers, Inga I Hitzeroth, Edward P Rybicki

**Affiliations:** 1 Biopharming Research Unit, Department of Molecular and Cell Biology, University of Cape Town, Rondebosch 7701, Cape Town, South Africa; ann.meyers@uct.ac.za (A.E.M.); inga.hitzeroth@uct.ac.za (I.I.H.); ed.rybicki@uct.ac.za (E.P.R.); 2 Institute of Infectious Disease and Molecular Medicine, Faculty of Health Sciences, University of Cape Town, Observatory 7925, Cape Town, South Africa

**Keywords:** African horse sickness, virus structure, replication, vaccine strategies

## Abstract

African horse sickness is a devastating disease that causes great suffering and many fatalities amongst horses in sub-Saharan Africa. It is caused by nine different serotypes of the orbivirus African horse sickness virus (AHSV) and it is spread by Culicoid midges. The disease has significant economic consequences for the equine industry both in southern Africa and increasingly further afield as the geographic distribution of the midge vector broadens with global warming and climate change. Live attenuated vaccines (LAV) have been used with relative success for many decades but carry the risk of reversion to virulence and/or genetic re-assortment between outbreak and vaccine strains. Furthermore, the vaccines lack DIVA capacity, the ability to distinguish between vaccine-induced immunity and that induced by natural infection. These concerns have motivated interest in the development of new, more favourable recombinant vaccines that utilize viral vectors or are based on reverse genetics or virus-like particle technologies. This review summarizes the current understanding of AHSV structure and the viral replication cycle and also evaluates existing and potential vaccine strategies that may be applied to prevent or control the disease.

## 1. Introduction

For several centuries, the devastating African horse sickness (AHS) has been a cruel scourge to horse owners in sub-Saharan Africa. The disease is infectious but non-contagious and causes high fatality rates in susceptible hosts. It is listed as a notifiable viral disease by the World Organization for Animal Health (OIE) because of its severity and the potential risk it poses for rapid global spread [1]. AHS remains the most economically significant equine disease worldwide.

The first known historical reference to AHS was recorded in an Arabian document entitled “Le Kitab El-Akoual El-Kafiah Wa El Chafiah”, which apparently relates to an epidemic that occurred in the Yemen in 1327 [2]. However, the virus is believed to have originated in Africa, with the first record of the disease on the continent being made by Father Monclaro in his account of the journey of Francisco Barreto to East Africa in 1569 [1]. Unlike zebras, which are endemic to the region, horses are not native to southern Africa and reference to AHS in South Africa was first made about fifty years after the introduction of horses and donkeys to the Cape of Good Hope by the early Dutch Settlers in 1657. A major outbreak occurred in 1719 when almost 1700 animals were reported to have succumbed to the dreaded “perreziekte” or “pardeziekte” [2]. Prior to 1953, periodic outbreaks seemed to occur at roughly 20–30 year intervals, the most severe being the outbreak in South Africa in 1854–1855, which claimed the lives of nearly 70,000 horses, more than 40% of the entire horse population of the Cape at the time [3]. Indeed in South Africa, the economic impact of the disease has been such that it directly and significantly influenced the progress and development of the field of veterinary science itself [4].

**Figure 1:**
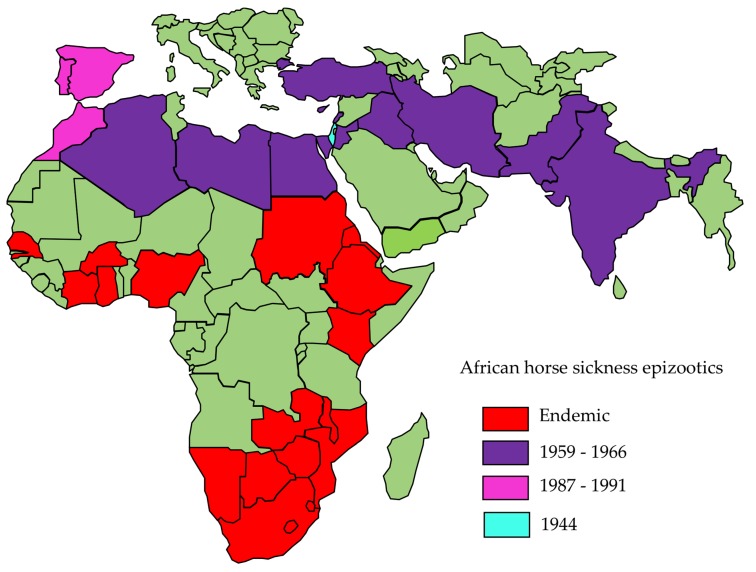
A map of African horse sickness outbreaks that have occurred worldwide during the last century.

AHS continues to occur regularly in southern African countries, but the virus has also occasionally escaped its geographical limitations and extended further afield to countries in North Africa, the Middle East, the Arabian peninsula, South-West Asia and the Mediterranean region (Figure 1) [5,6,7]. The severe epizootic in the Middle East and South West Asia between 1959 and 1963 was responsible for the deaths of over 300,000 equines and was finally only arrested as a result of a concerted vaccination campaign and widespread depletion of susceptible animals [7,8]. AHS-free countries with milder climate conditions are believed to be increasingly at risk for outbreaks of the disease due to the northward migration of the midge vector as a result of global warming and climate change [9,10,11]. Such an AHS outbreak in Europe would have significant economic and emotional consequences for horse owners on the continent, indicating the pressing need to develop new, safe, efficacious and cost-effective vaccines which would additionally allow differentiation between vaccinated and infected animals (DIVA). Such vaccines would not only address the concerns of the South African equestrian community but would also serve as acceptable prophylactic or rapid response vaccines in the European and other emerging outbreak contexts.

## 2. African Horse Sickness Virus

The first sign that the causative agent of AHS may be a virus was provided by M’Fadyean [12], who demonstrated the filterability of the infectious organism by successfully transmitting the disease using a bacteria-free blood filtrate from an infected horse. This finding was later confirmed by Theiler and Nocard, who concluded that the disease was caused by a virus [2]. Further research done by Theiler led to the suggestion that more than one strain of the virus may exist, and that acquired immunity against one strain would not necessarily afford protection against a different heterologous strain [3].

It is now known that the disease is caused by nine distinct serotypes of African horse sickness virus (AHSV) [13,14], a virus with a multicomponent linear double stranded RNA (dsRNA) genome belonging to the *Orbivirus* genus of the family *Reoviridae* [1,15]. The idea that AHSV may possibly be transmitted by hematophagous arthropods of the *Culicoides* genus was first suggested by Pitchford and Theiler in 1903 [3]. Du Toit [16] subsequently demonstrated that mixed pools of wild-caught *Culicoides* species were infected with AHSV. This was confirmed by Mellor, et al. [17] and Boorman, et al. [18], who demonstrated the occurrence of AHSV replication within a *Culicoides* species after oral ingestion. Field and laboratory-based trials have implicated *C. imicola* and, to a lesser extent, *C. bolitinos* as the primary vectors of AHSV, although some evidence does exist for possible AHSV transmission by other arthropod vectors [19]. The ability of AHSV to propagate in both arthropod and mammalian cells is a notable feature shared with all orbiviruses, and one which distinguishes them from some other members of the family *Reoviridae* [20].

Zebra are generally resistant to AHS and have been identified as asymptomatic maintenance hosts of AHSV, while mules and donkeys are much less susceptible than horses to the disease [21]. Dogs are the only non-equine animal species that have been shown to contract AHS, with evidence suggesting that the route of infection may, although not exclusively, be via the ingestion of infected meat [22,23]. However, dogs do not appear to be important hosts for AHSV, most likely due to the fact that they are not preferential feeding targets of the midge vector.

**Figure 2:**
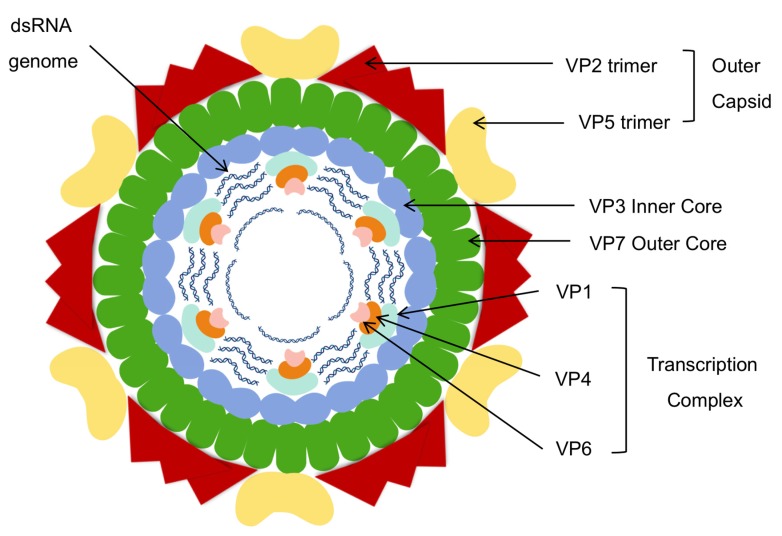
Schematic representation of the AHSV virion. The genome contains 10 segments of linear dsRNA coding for 12 proteins. The virion is non-enveloped with a triple capsid structure and is about 80 nm in diameter, enclosing the genome and transcription complexes. The inner core layer has T = 1 symmetry with each of the 60 units composed of a homodimer of VP3, while the outer core is composed of 260 trimers of VP7 and has T = 13 icosahedral symmetry. The outer capsid layer consists of 120 globular trimers of VP5 and 60 triskelion-shaped spikes of VP2. *Image created with Biorender.com*.

The African horse sickness virion is a structurally complex and highly organized non-enveloped isometric particle with a diameter of ±80 nm [24,25]. Like the genus prototype bluetongue virus (BTV), with which it is morphologically almost identical, the non-enveloped virion is quasi-icosahedrally symmetrical and is composed of three concentric protein layers [26,27,28]. The innermost layer encloses the AHSV genome, which consists of 10 segments of linear dsRNA, encoding seven structural (four major and three minor) and five non-structural proteins [29]. Two of the major structural proteins, VP5 and VP2, make up the outer capsid layer, while the other two major structural proteins VP3 and VP7, and the three minor structural proteins, VP1, VP4 and VP6, make up the AHSV core particle (Figure 2).

In recent years, considerable advances have been made in determining the BTV atomic structure and mechanism of assembly as well as the functions of the individual protein components of the viral particles [20,30,31,32,33]. This has recently been clearly and concisely reviewed elsewhere [34]. Less extensive studies have been conducted on the molecular biology of AHSV, but the morphological and biochemical similarity between BTV and AHSV is indicative of a similar mode of replication and assembly for both viruses.

The inner core layer of AHSV is composed of 60 asymmetric dimers of protein VP3 (103 kD), the most conserved protein among the different serotypes [35]. Each dimer consists of two VP3 isoforms comparable to the A and B conformations of BTV VP3 [31] and the inner core is thus completed by the assembly of 12 decamers, each of which consists of five copies of the VP3 (A) molecule with five VP3 (B) molecules in between. This architecture is not in agreement with the hypothesis for icosahedral symmetry proposed by Caspar and Klug [36], but a model of geometrical quasi-equivalence has been proposed whereby the symmetry of the VP3 layer is T = 2 rather than T = 1 [31]. The thin VP3 shell thus defines the overall shape and size of the viral particle and provides a scaffold for the deposition of the outer core and capsid protein layers [37].

In both BTV and AHSV, the minor structural proteins VP1 (RNA-dependent RNA polymerase, 150kD), VP4 (capping enzyme, 78kD) and VP6 (helicase and ATPase, 36kD) form 12 flower-shaped transcription complexes (TC) attached to the VP3 layer directly under each of the fivefold vertices [31,33,38]. Internal concentric layers of RNA comprising the full dsRNA genome are situated around these transcription complexes in shallow grooves in the inner VP3 surface [37,39].

BTV contains pores in the VP3 layer at the fivefold axes which are lined with four arginine (Arg) residues that are conserved across all the serotypes, and which are believed to play a role in electrostatic steering of RNA entering or leaving the sub-core [31]. In the 3D image construction of AHSV, these pores were closed [38], but the same 4 Arg residues are strictly conserved across all 9 AHSV serotypes. It is possible that, as has been shown to be the case for BTV [40], these pores may be enlarged by activation of VP1 during transcription, permitting the exit of nascent mRNA.

The AHSV core is made rigid by the addition of 780 monomers of protein VP7 (38 kD) which is highly conserved across the serotypes and is the group-specific antigen currently used in AHSV ELISA-based diagnostic tests [41]. The atomic structure of BTV VP7 has been determined by x-ray crystallography [42], but only the upper domain of AHSV VP7 has been crystallized [43], as the protein was unfortunately cleaved in half during the crystallization process. The VP7 monomers contain a helical lower domain and an upper anti-parallel-ß-sandwich domain [44]: these trimerize in solution by twisting around each other so that the top domain of one monomer rests on the lower domain of the adjacent VP7 subunit.

These 260 VP7 trimers conform to the principle of quasi-equivalence as, although they are chemically identical, five different trimer types can be identified based on slightly different side chain arrangements. These are named P, Q, R, S and T, denoting their position with regard to the five-fold vertices. They crystallize perpendicularly onto the VP3 sub-core, making thirteen unique contacts so that each icosahedral subunit contains a P, Q, R and S trimer and one monomer of a shared T trimer located on the adjacent three-fold axis [31,45].

The trimers are robust building blocks, which also make extensive connections between each other [42]. They are arranged as either six-member rings, or five-member rings at the fivefold vertices, thus forming 132 channels over the core particle surface. Symmetries between the inner and outer core layers are best matched at the three-fold axes and, as the T trimers situated here also seem to be the most tightly attached, it has been suggested that these are probably the first set of trimers to attach to the VP3 layer while the P trimers, which are more loosely attached, assemble last [46].

An interesting phenomenon which distinguishes AHSV VP7 from BTV VP7 is the fact that despite the 70% amino acid sequence homology [47], BTV VP7 is soluble while AHSV VP7 is not. AHSV VP7 is, in fact, highly hydrophobic, and the VP7 trimers have been shown to aggregate into flat hexagonal crystals up to 250 um in length and up to 25 um wide, both when expressed in insect cells via baculovirus-mediated expression and in naturally infected mammalian cells [41,48].

The viral particle is completed by the addition of 120 globular trimers of VP5 (57 kD) and 60 triskelion-like VP2 (123 kD) spikes which together form the outer capsid layer. VP2 is the most variable protein among the serotypes and contains the antigenic determinants which elicit serotype-specific neutralizing antibodies [49]. The viral particles contain 180 monomers of VP2, each of which contains a hub, body, hairpin and tip domain, the latter containing the neutralizing antibody binding sites [30,38]. Cryo-EM and 3D reconstruction have revealed that the VP2 spikes are formed by trimerization of the hub domains of three VP2 monomers. The base of each VP2 spike interacts with a Q type VP7 trimer, while the body domains of the three VP2 monomers connect with a P, R and S - type VP7 trimer, respectively. Furthermore, for BTV, Zhang et al. identified a zinc finger motif and a putative sialic acid (SA) binding domain in the VP2 trimer hub [32].

The VP5 trimers exist as two quasi-equivalent conformers and are positioned between the propeller-like arms of the VP2 triskelions, bridging the 120 channels formed by the six-member VP7 trimer rings. The viral outer shell therefore covers nearly all of the inner shell, but significantly, leaves the 20 VP7 T trimer spikes on the icosahedral three-fold axes accessible to possible antibody binding [50].

In addition to the seven structural proteins that make up the viral particles, five non-structural proteins, NS1, NS2, NS3, NS3a (lacking 13 N-terminal amino acids) and NS4, are also synthesized in infected cells. These are involved in virus replication, assembly and transport from infected cells [29]. The tubular structures commonly observed in the cytoplasm of infected cells are composed of NS1, which has been shown to play a role in the preferential up-regulation of viral protein synthesis [51]. The single-stranded RNA (ssRNA)-binding protein NS2 forms viral inclusion bodies (VIBs), which recruit viral ssRNA and form a scaffold for viral replication and core assembly [52,53]. Viral particle release is mediated by NS3/NS3a, the only AHSV glycosylated membrane protein. This, unlike BTV NS3/NS3a, which is highly conserved, is the second most variable protein across the different serotypes. Once the outer capsid proteins VP2 and VP5 have been acquired by the newly formed cores, NS3 facilitates egress of fully formed viral particles from infected cells [54,55]. The most recently identified non-structural protein, NS4, is thought to play a role in modulating host innate immunity by counteracting the interferon response in infected cells [56,57].

## 3. Viral Infection and Replication

AHSV host infection is believed to be initiated by the outer capsid protein VP2, the host cellular surface receptor and capsid protein VP5, which contains a characteristic coiled-coil motif typical of membrane fusion proteins [32,58]. Due to the location of the putative sialic acid (SA) binding domain in the VP2 trimer hub, Zhang et al. have postulated that the VP2 trimers may attach to the surface of cells through two different interactions [32]. First, the antigenic tip domains bind to certain surface cell receptors which have yet to be identified. There is some preliminary evidence implicating heparin sulphate as one possible candidate [59], but due to the high sequence variability in the AHSV outer protein VP2, it is possible that different receptors and entry mechanisms may be utilized by the different serotypes to enter distinct cell types [60]. These initial bonds are then stabilised by a second connection between the SA-binding domain and a surface glycoprotein such as glycophorin A [61,62], which is a heavily glycosylated sialoglycoprotein abundantly present on the surface of equine erythrocytes. The sensitivity of VP2 to serum proteases has been established [48,63], and it has been suggested [38] that a central domain at the top of the AHSV VP2 triskelion hub, which is absent in BTV VP2 trimers, could be the target site for such a horse serum protease. This domain is situated directly above the BTV putative SA-binding site and cleavage of AHSV VP2 in this region would thus increase accessibility to this potential binding site.

Due to the structural similarity between the two viruses, a model for the entry of AHSV into the host cell can be derived from the current understanding of BTV infection. The process is believed to be initiated by proteolytic cleavage of VP2 either in infected insect saliva or in the host serum, after which the virion attaches to the host cell membrane [64]. These initial studies suggested that viral cell entry was accomplished by clathrin-mediated endocytosis. However, more recently evidence has been presented which describes a macropinocytosis-like entry route dependant on actin and dynamin [60]. In both instances, the low pH (6.0–6.5) within the early endosome disturbs the interactions between VP2 and VP7, facilitating detachment of the VP2 trimers and disrupting the zinc finger motif situated at the interface between the VP2 hub and body domains, which is believed to play a role in controlling conformational changes [30]. Together with a further lowering of the pH (~ 5.0) in the late endosome, the removal of VP2 causes a re-folding of VP5, which in turn leads to the outward protrusion of barb-like structures from the particle surface with the VP5 protein trimers remaining tethered to the particle by their anchoring domains [32,65]. These barb-like fusion peptides insert themselves into the endosomal membrane, causing release of the viral core particle into the cytoplasm.

The removal of the outer capsid proteins, and the release of viral cores into the cellular environment containing the necessary host substrates and transcription factors causes the core to become transcriptionally active. Each of the gene segments is then simultaneously and repeatedly transcribed by VP1 to produce ssRNAs [30,66], which are modified by the capping and methylation activity of enzyme VP4 within the core before being released into the cytoplasm [67]. The viral dsRNA is thus kept within the core particle protected from detection by components of the host cell innate immunity. The nascent ssRNAs act as mRNAs for the synthesis of viral proteins using the host cell machinery and later, in the newly formed cores, as templates for dsRNA gene synthesis. The VIBs act as the sites of viral assembly and protein NS2 plays a role in directly and specifically sequestering the 10 ssRNAs, together with the three enzymatic proteins VP1, VP4 and VP6 and inner core protein VP3, for encapsidation and the formation of new sub-core particles [53,68].

**Figure 3:**
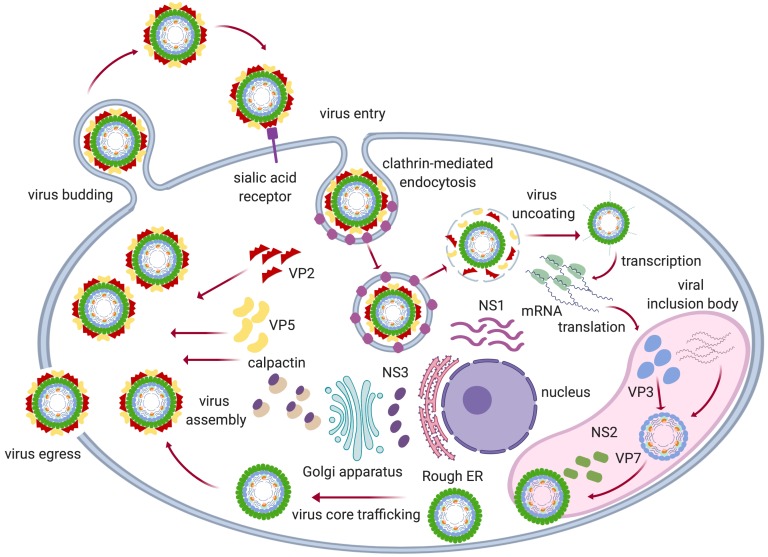
Diagrammatic representation of the replication cycle of BTV/AHSV. The virus enters the cell by the attachment of VP2 to sialic acid receptors and either clathrin-mediated endocytosis or macropinocytosis. The acidic pH in the endosome causes the loss of VP2 and mediates VP5 membrane permeabilization, which results in uncoating of the virion and release of the transcriptionally active core particle into the host cell cytoplasm. Transcription and translation of viral proteins occurs, utilizing the host cell machinery and the VIBs act as sites of assembly for the progeny virions. Assembled core particles are then trafficked from the VIB on exocytotic vesicles by NS3 interaction with calpactin. The outer capsid proteins VP5 and VP2 are acquired during this process to produce mature virions. Particles are released from the cell via budding mediated by NS3 or via host cell lysis. *Image adapted from* [53] *and created with Biorender.com*.

The deposition of VP7 trimers serves to stabilize the particles, and phosphorylation of NS2 then regulates their exit from the VIBs in order to acquire the two outer capsid proteins VP5 and VP2 for the formation of mature progeny virions [69,70]. Although the release of these virions from infected mammalian cells is predominantly affected by cell lysis accompanied by significant cytopathic effects, the viruses have also been shown to use a budding mechanism for viral egress earlier on in the infection cycle. The latter is mediated by utilising the host exocytosis pathway and the membrane destabilising action of non-structural glycoprotein NS3, which functions as a viroporin and also interacts with calpactin to function as a bridging molecule between the new virions and the host cell export machinery [71,72]. In the insect vector, where the establishment of a persistent viral infection is important, there is no observable cytopathic effect and viral release is mediated exclusively via vesicle formation at the cytoplasmic membrane [53] (Figure 3). It is interesting to note that the more variable AHSV NS3 causes a much greater cytopathic effect (CPE) earlier on in the infection cycle than BTV NS3, possibly indicating that AHSV NS3 is expressed at a higher level or is more toxic than BTV NS3 [73].

Although the primary route of BTV and AHSV host infection is believed to be initiated by the outer capsid proteins, there is evidence to suggest that BTV core-like particles (CLPs), i.e., particles that have lost the outer capsid proteins, are also able to infect both insect and, to a lesser extent, mammalian cells [74]. Interestingly, in this regard, the upper domains of both BTV and AHSV VP7 trimers have characteristic Arg-Gly-Asp (RGD) motifs, albeit in slightly different locations [43]. RGD domains in biological systems are associated with integrin-ligand recognition and fusion of molecules to cell membranes [75]. The fact that there are holes in the surface of the outer capsid layer of both BTV and AHSV particles makes it tempting to speculate that these RGD sites on VP7 may play a role in the ability of viral CLPs to infect cells.

## 4. African Horse Sickness Disease

Three distinct forms as well as a mixed form of AHS have been described. However, it is possible that these represent points on a continuum of virulence as some disease outbreaks are characterized by only one form of the disease, while during other outbreaks, multiple forms occur [76]. The most severe form of AHS, with mortality rates exceeding 95%, is the pulmonary form or “dunkop” (thin head). This is an acute febrile disease accompanied by mild depression, sweating, spasmodic coughing, anorexia and respiratory distress, with a possible frothy nasal discharge in the terminal stages [1,3]. The cardiac form or “dikkop” (thick head), with a mortality of about 50%, is characterized by fever, swelling of the head, neck and supraorbital fossae and sometimes, petechial hemorrhages in the eyes. The mildest form of AHS is generally not fatal and is accompanied by a low-grade fever, often more pronounced in the afternoon, anorexia, depression and congestion of the mucous membranes. The most common form, with a 70% mortality rate, is a mix of the pulmonary and cardiac forms.

The bite of an infected *Culicoides* midge signals the potential initiation of infection in a susceptible host, after which initial AHSV replication occurs in the regional lymph nodes. This primary viraemia is responsible for disseminating the virus to all parts of the body [1]. Viral particles are known to associate with erythrocytes and monocytes and are transported in the bloodstream to the endothelial cells of the lungs, spleen and other lymphoid tissue, which are the main sites of secondary replication [76,77]. Although the level of replication is relatively low in these organs, the virus causes severe injury to the endothelial cells and the symptoms of oedema and pleural effusion, which characterize the severe form of AHS, are believed to be the result of increased vascular permeability and the impairment of circulatory and respiratory systems.

The primary factors which influence the severity and duration of the disease in horses are related to the virulence of the virus and the immune status and susceptibility of the animal. Host genetics must play a role, as is evidenced by the susceptibility of both horses and zebra to AHSV, yet only horses contract AHS disease. An animal which has recovered from a prior infection is fully protected by re-infection with the same serotype and may well only contract a fever or the cardiac form of AHS upon exposure to a heterotypic viral strain. However, Erasmus [78] noted that excessive vaccine administration may lead to an immunological unresponsiveness or even hypersensitivity.

Virulence is related to tissue tropism, and it would seem that the virus itself is the primary determinant of the resultant form of disease [79]. AHSV field isolates are believed to be composed of a mixed virus population with regard to the host tissue target, and the virulence of the particular isolate may thus be determined by the percentage of particles affecting vital organs, such as the lungs, for example. In this sense, Erasmus [79] suggests that viral attenuation may be the result of specific selection of viruses which do not have affinity for the tissues of vital organs.

Alexander [80] was the first to demonstrate the humoral nature of the AHSV immune response when he utilized a mouse neutralization test to indicate a strong association between the production of neutralizing antibodies and protective immunity. The antigenic determinants responsible for this neutralising antibody response are located on capsid protein VP2: several studies have suggested putative sites for these epitopes, mainly in the 5′ terminal half of the protein [38,49,81,82,83,84]. However, the probable contribution of cell-mediated immunity cannot be ignored—at least three CD8 T-cell epitopes have been identified on VP2 or NS1 [85] and others have reported the stimulation of IFN-y responses in vaccinated animals [86,87]. The degree to which the presence of neutralizing antibodies may be regarded as an adequate correlate of protection therefore remains to be established.

African horse sickness is on the OIE list of notifiable viral diseases, which means that it is compulsory for member states like South Africa to inform the organization of any change of disease status. In South Africa, the Western Cape Province has historically been relatively free of AHS; in order to maintain the country’s horse export status, an AHS controlled area—to and from which the movement of all horses is strictly monitored—was established in 1997 [88]. The area consists of an AHS-free zone, which is the small Cape Metropolis where no cases of AHS have ever been recorded, a surrounding surveillance zone and beyond that, a zone of protection. Whenever a new outbreak occurs in the surveillance zone, horse exports to the EU are suspended for a minimum of 2 years.

According to the EU surveillance requirements, every month, at least 60 identified unvaccinated horses distributed throughout the free and surveillance zones are serologically tested for AHSV [89]. Local law requires all suspected cases of AHS to be reported to the state veterinary authority, and all equine deaths due to AHS undergo official equine necroscopy examination. As a further measure of control, it is compulsory to obtain permission prior to vaccinating horses with the AHS polyvalent live attenuated vaccine (LAV) produced by Onderstepoort Biological Products (OBP, Pretoria) in the free and surveillance zones.

The clinical presentation of AHS is often sufficient to make a tentative diagnosis of the disease; however, particularly in the early stages, differentiation from other equine diseases, such as equine encephalosis (EEV), equine viral arteritis (EVA) and West Nile virus (WNV), may not be possible. Traditional methods of virus isolation and serotyping by virus neutralization assay can be used to make a definitive diagnosis, but these tests rely on the availability of appropriate reference strains and antisera and can take weeks before a result can be obtained. They are therefore unsuitable for early detection of AHS disease as often the animals will die before a detectable humoral response is raised [90]. The currently used indirect ELISA test is based on detection of the group-specific VP7 antigen in the serum sample [91]. However, although accurate, it is neither possible to determine the virus serotype nor to differentiate between vaccinated and infected animals using this method.

Over the last two decades, several real-time reverse transcription polymerase chain reaction (RT-PCR) assays have been developed and made available to the scientific and veterinary communities [92]. Some tests were group-specific [93,94,95,96], based on amplifying AHSV VP7 or one of the non-structural proteins and two of these are recognized as official screening tests by the OIE [97]. The type-specific (TS) tests generally require separate PCR assays to diagnose each of the nine serotypes [90,98,99], but the most recently described test was designed such that three serotypes can be detected in the same assay [92]. These triplex AHSV TS RT-quantitative PCR assays can be directly applied to nucleic acid extracted from blood samples from AHSV-infected horses, meaning that samples can be extracted and evaluated within 4 h of their arrival at the laboratory. The AHSV serotype in a field outbreak can thus be rapidly determined, making this test most useful in directing the timeous implementation of appropriate vaccination and control strategies.

## 5. Prevention and Control

There is no cure for AHS and no specific treatment aside from rest and good animal husbandry. Various interventions, such as non-steroidal anti-inflammatory drugs for alleviating pain and reducing fever, antimicrobials to fight secondary bacterial infection or corticosteroids to help stabilize cell membranes and preserve vascular membrane integrity, have been employed, but all these treatments are supportive rather than curative (African Horse Sickness Trust). Anecdotal reference to homeopathic remedies has also been made, but there is no scientific evidence to prove the efficacy of such treatments in AHS cases. The implementation of certain husbandry modifications, such as stabling animals before dark in vector-proof housing, using insect repellants and encouraging natural vector predators like fish, frogs and bats, may assist in prevention [1,100]; however, ultimately, vaccination of animals remains the most successful method of prevention and control.

### 5.1. Live Attenuated Vaccines

Alexander [101] was the first to demonstrate that a mouse-adapted strain of AHSV could be propagated in chicken embryos, and that serial passage in embryonated hens’ eggs caused attenuation of the virus without loss of immunogenicity. His studies also supported the existence of multiple strains of the virus, as he found a large variation (26%–81%) in the number of horses that were protected after being challenged a second time with a different virus isolate. The successful propagation of AHSV in mammalian tissue culture by Erasmus [102], aided by the discovery that viral plaque size could be used as a genetic marker to identify avirulent clones of AHSV from a mixed population [78], further advanced the development of live-attenuated vaccine (LAV) strains. The polyvalent LAVs that have been successfully used to vaccinate horses over the past six decades are based on these viral formulations. However, although the frequency and severity of outbreaks has declined significantly since these vaccines have come into use, many horse deaths due to AHS still occur in South Africa every year.

The currently used LAV is supplied in two polyvalent vials containing 3 (serotypes 1, 3 and 4) and 4 (serotypes 2, 6, 7 and 8) AHSV serotypes each. Neither AHSV 5 nor AHSV 9 are included in the vaccine [103]: serotype 5 was originally included but was withdrawn in 1990 following reports of residual virulence, believed to be the result of re-assortment between serotypes 4 and 5 in the vaccine formulation [104]. Serotype 9 has never been included due to its low incidence in southern Africa and, because cross protection between serotypes 1 & 2, 3 & 7, 5 & 8 and 6 & 9 has been documented [3,78,104], protection against AHSV 9 is expected to be provided by AHSV 6. There is no cross-protection between AHSV 4 and any of the other serotypes. Of concern, however, is the fact that in 2006 both AHSV 5 and 9 dominated outbreaks in South Africa, particularly in the Western Cape Province: this raises questions about the competency of the LAV to provide sufficient protection against these two AHSV serotypes.

Although there is little argument that the LAV is still the best option in the fight against AHS, its use has raised concerns with regard to other important issues. The serotype-specific immune response within horse populations, as well as between the different serotypes appears to be quite variable, and it may take as many as 8 vaccination courses over 6 years before an animal is fully protected against all nine AHSV serotypes [104,105,106]. Furthermore, gene segment re-assortment between outbreak and vaccine strains may lead to the establishment of new genetic variants or reversion to virulence of attenuated vaccine strains [1]. Indeed, a study comparing the whole genome sequences from AHSV isolates responsible for outbreaks between 2004 and 2014 in the controlled area of the Western Cape with LAV and AHSV reference strains demonstrated conclusive evidence of re-assortment between and reversion to virulence of viruses within the LAV itself [88]. The outcome of this study highlights the importance of employing judicious LAV vaccination strategies and genetic screening of circulating field strains during AHS outbreaks.

Another shortcoming of the LAV is the inability to serologically differentiate vaccine-induced immunity from that induced by natural infection, i.e., the absence of what is known as DIVA capacity. This differentiation is important both for early detection of disease and sero-surveillance and can also limit unnecessary culling of animals in an outbreak situation. A further major issue regarding routine vaccination with the LAV is the fact that it is not licensed for use outside of the African sub-continent, which has a hugely negative impact on the international equine trade and export industry.

### 5.2. Inactivated Vaccines

Inactivated or “killed” vaccines have been prepared by treating mammalian cell-cultured AHSV with formaldehyde or ß-propiolactone [107] or with bromoethylenimine [108]. The latter acts on nucleic acid but not protein, ensuring that the immunogenic properties of the vaccine are not compromised. An inactivated AHSV 9 vaccine tested in guinea pigs and horses [109] elicited a comparable neutralizing antibody response in both animal species, confirming the usefulness of the guinea pig as a small animal model to test the efficacy of potential vaccine candidates [110]. The vaccine proved to be safe and all horses survived a challenge with the same virus used to generate the killed vaccine.

A formalin-inactivated AHSV vaccine was commercially produced and used during the 1987–1991 AHS outbreak in Spain, Portugal and Morocco, but although it proved to be efficacious at the time, this vaccine is no longer available [1]. The main drawbacks with regard to inactivated vaccines are firstly that they are expensive to produce, requiring large-scale isolation of infectious virus, which poses a significant bio-containment risk; secondly, repeated inoculations may be required to ensure long-lasting protective immunity. Furthermore, although the risk of gene segment re-assortment and reversion to virulence are mitigated with this type of vaccine, differentiation between vaccinated and infected animals is not possible.

### 5.3. Recombinant Vaccines

Due to raised international awareness and local dissatisfaction with the current vaccine, in recent years, AHSV research has focussed on the development of recombinant vaccines. These have largely been based on producing the antigenic AHSV proteins involved in eliciting a protective immune response, particularly the outer capsid proteins VP2 and VP5, and investigating the best ways in which to present them to the host’s immune system.

#### 5.3.1. DNA Vaccines

Besides the AHSV proteins themselves, there has been some investigation of the efficacy of using naked AHSV VP2 DNA as a vaccine candidate [111]. Although a VP2-specific humoral and cellular immune response following inoculation of a single horse was observed, and the same horse survived an AHS outbreak during the following rainy season, the neutralizing antibody titre reported was sub-optimal and no experimental challenge ensued. Furthermore, vaccination of hens with cloned VP2 cDNA stimulated the production of egg yolk IgY antibodies with a serum neutralization titre 80-fold less than that obtained following vaccination with purified AHSV. The potential for producing a suitable AHSV DNA vaccine thus seems limited.

#### 5.3.2. Subunit Vaccines

Recombinant AHSV VP2 produced via the baculovirus expression system has been used either singly or in combination with VP5 and/or VP7 as a subunit vaccine, and was shown to induce protective immunity against the virus [112,113,114]. However, recombinant soluble antigens are generally poorly immunogenic and the aggregation of baculovirus-expressed VP2 purified from insect cell lysates together with the requirement for repeated boost inoculations [115], and the use of potent adjuvants to enhance immunogenicity [116] have limited the usefulness and application of this type of vaccine. Furthermore, although subunit vaccines are advantageous in that they permit differentiation between AHSV-vaccinated and infected animals, baculovirus expression requires growth under sterile conditions and is uneconomical for an animal vaccine due to the high cost of media required to culture insect cells.

#### 5.3.3. Poxvirus-Vectored Vaccines

Poxvirus vectored vaccines are recombinant poxvirus strains which have been genetically modified to contain a copy of the gene of interest within the viral genome. The vaccine is delivered directly to the cells where the viral protein is expressed and presented to the host immune system for the stimulation of both humoral and cellular immunity.

Vaccination of horses with an adjuvant-formulated canarypox vaccine (ALVAC-AHSV) expressing both AHSV 4 VP2 and VP5 was shown to elicit serotype-specific neutralizing antibodies against the virus [117]. Upon challenge, horses which received a sufficiently high dose of vaccine developed sterilizing immunity against AHSV 4 with serum neutralization titres ranging from 10 to 80 (expressed as the reciprocal of the highest dilution that provided >50% cell protection). However, stimulation of neutralizing antibodies has yet to be established as a definite correlate of protection: this was emphasized by the survival of a seronegative horse after challenge with virulent AHSV. Furthermore, in a follow-up study using the same vaccine, gamma interferon-producing cells were detected after stimulation with both VP2 and VP5, indicating that cell-mediated immune responses most likely also played a role in protecting against the viral challenge [86].

Since 2009, several studies on the development of an alternative AHSV candidate vaccine based on a modified vaccinia Ankara (MVA) virus have been published [85,118,119,120,121,122,123]. The MVA strain was originally produced by extensive passage of the chorioallantosis vaccinia virus Ankara (CVA) in chicken embryo fibroblast cells (CEF). This resulted in the loss of 12% of the viral genome, including genes that interfere with the host immune response and caused inability to replicate in most mammalian cells [124]. However, replication is only blocked after DNA synthesis, so gene expression still occurs, with resulting expression of recombinant antigens inside infected cells. Recombinant MVA vaccines are therefore non-replicative live viral vectors that can induce both a humoral response as well as stimulate T-cell immunity by facilitating intracellular presentation of the antigen of interest via MHC molecules on the cell surface. Interestingly, MVA vaccines have been shown to be most effective in prime-boost regimens, i.e., when administered as a heterologous boost following a strong prime vaccination [125]. Boosting of an existing T-cell response to a recombinant antigen causes an amplification of the initial response and reduces the response to antigens on the viral vector itself, thus avoiding the issue of pre-existing immunity to the viral vector and allowing re-use of the vaccine.

However, homologous prime-boost vaccination with MVA expressing AHSV VP2 (MVA-VP2) has also been shown to elicit neutralizing antibodies which provided complete protection in both mice and horses [118,119,120,121]. The type 1 interferon receptor knockout (IFNAR -/-) mice used in these experiments were chosen as small animal challenge models as AHSV infection causes similar clinical pathologies and mortality rates to those observed in horses. In these studies, protection was provided by vaccination with MVA vaccines expressing only outer capsid protein VP2, indicating that VP5 is not essential for, but may improve vaccine efficacy.

The role of cell-mediated immunity appears to be less important than antibody responses, as the transfer of splenocytes from MVA-VP2-vaccinated mice to unvaccinated mouse recipients did not cause a statistically significant reduction in viraemia [123,126]. Furthermore, passive immunization with vaccinated donor sera protected recipient mice from infection, demonstrating the primary protective role of the neutralizing antibody response. However, cell-mediated immunity is likely to play an additional protective role to some extent, as mice immunized with either MVA-VP2 or MVA-NS1 have been shown to develop gamma-interferon-producing cells when stimulated with peptide sequences on VP2 and NS1 [85].

In a study to investigate a polyvalent AHSV vaccination approach, horses were immunized and boosted four weeks later with either MVA-VP2(4) or MVA-VP2(9) or both, simultaneously or sequentially [119]. Simultaneous vaccination with recombinant MVA-VP2 of both serotypes, induced a statistically significant virus neutralizing antibody (VNAb) response against AHSV 4 and AHSV 9 as well as a cross-protective response to AHSV 6 in the horses which received MVA-VP2(9). Furthermore, four months later, when the VNAb titres had decreased dramatically, vaccination with MVA-VP2(5) representing a third AHSV serotype, not only elicited VNAb against AHSV 5, but also induced an anamnestic response towards AHSV 4, 6 and 9 as well as the cross-reactive AHSV 8. These results demonstrate the suitability of MVA-VP2 to be used as a polyvalent vaccine mixture providing protection against more than one AHSV serotype. The results also suggest the possibility that other sub-dominant cross-reactive epitopes may exist between AHSV serotypes 5, 6, 8 and 9. This study further confirms that any possible pre-existing immunity to the viral vector does not impact negatively the usefulness of the MVA-VP2 vaccines.

The immune responses induced by four different AHSV 4 MVA-VP2 vaccines, namely live MVA-VP2, heat, inactivated MVA-VP2, UV, inactivated MVA-VP2 and sucrose gradient-purified MVA-VP2, proved that both pre-formed VP2 in the MVA vaccine and transient expression of VP2 in the vaccinated host’s cells contribute to inducing a protective immune response [122]. The inactivated MVA-VP2 vaccines, containing only pre-formed VP2, induced lower VNAb titres than the live MVA-VP2, yet the gradient purified MVA-VP2, containing no pre-formed VP2, also induced a weaker immune response than that induced by live MVA-VP2. In fact, sterilizing immunity was only induced by the live MVA-VP2 vaccine. It is likely that the transient intracellular expression of conformationally intact VP2 in infected cells activates T-cells, lending credence to the possible supportive role of cellular-mediated immunity in the protective immune response.

#### 5.3.4. Reverse Genetics Vaccines

Over the last decade, reverse genetics systems have been used to generate novel live virus-based BTV and AHSV vaccine candidates, engineered according to a rational design rather than by random serial passage attenuation [127,128,129,130,131,132,133,134,135,136,137,138]. These live vaccine strains depend on the availability of cloned cDNA copies of the viral genes and are produced in mammalian cell lines via a double transfection strategy. Firstly, a primary viral replication complex is pre-expressed by transfection with expression plasmids encoding the five viral proteins, VP1, VP3, VP4, VP6 and NS2. A second transfection with ten exact copy capped T7 viral RNA transcripts, which serve as effective substitutes for authentic core-derived viral transcripts, then triggers full replication and enables virus rescue. Different AHSV serotypes can be rescued by using the same primary transcription complex, and then exchanging the T7 RNA transcripts of one or more capsid proteins [130]. More importantly for vaccine purposes, genes encoding these proteins can be incorporated into a common viral genome which has been precisely engineered to contain one or more defective genes.

Two main vaccine platforms to produce defective virus strains have been developed using this technology. Entry Competent Replication Abortive (ECRA) vaccine strains, previously also referred to as Disabled Infectious Single Cycle (DISC) vaccines, lack a functional VP6 gene and are therefore unable to complete even a single replication cycle in infected cells [127,131]. The defective vaccine is rescued and propagated in a complementary cell line expressing VP6 *in trans* and viral antigens capable of eliciting the expected antibody response are expressed in normal cells, but no active infection ensues. In contrast, Disabled Infectious Single Animal (DISA) vaccine strains lack a functional gene for expression of non-essential non-structural protein NS3/NS3a [129,132,135]. The absence of these proteins prevents viral egress, thus inhibiting viraemia and allowing only local replication in infected cells, with no propagation in nor transmission by midges. Both these vaccine candidates fulfil the criteria for DIVA compliance, as antibodies to the missing viral protein in each case would be absent in vaccinated animals but present in animals which have been infected.

The main goal in new AHSV vaccine development is to provide protection against all nine serotypes of the virus. Initially, an attempt was made to develop a set of defective AHSV virus strains, each consisting of a common core coated with a different serotype-specific outer capsid protein VP2 [129]. However, the exchange of only a single protein resulted in unequal and significantly lower re-assortant viral titres compared to the parental virus strain. To produce suitably-replicating defective vaccine strains for each serotype, it appears necessary to exchange between two (VP2 and VP5) and five (VP2, VP3, VP5, VP7 and NS3) proteins on the common backbone, the number depending on the desired viral serotype [127]. The safety and immunogenicity of both monospecific (AHSV 4) and multivalent cocktail (AHSV 1/4/6/8) ECRA vaccines was tested in ponies; these were protected against virulent challenge with AHSV 4 [131]. Pre-challenge serum neutralization titres were in the range of 8–64 (expressed as the reciprocal of the highest dilution that provided >50% cell protection), below those generally obtained following vaccination with the AHS LAV [106], but nevertheless demonstrating the potential efficacy of a reverse genetics vaccine candidate to protect against the disease. Although the technology looks promising, further research is necessary to determine the minimum dose requirement and longevity of the immune response. Furthermore, the associated cost and upscaling requirements may deter successful commercialization of these potential vaccine candidates.

#### 5.3.5. Virus-Like Particle Vaccines

Virus-like particles (VLPs) are safe, non-replicating protein complexes which mimic the structure of intact virions. They possess self-adjuvanting properties and have the advantage of being highly immunogenic compared to subunit vaccines, as epitopes are displayed in ordered repetitive arrays on the particle surface [139]. The size of VLPs ensures appropriate drainage into the lymph nodes and is also optimal for uptake by antigen-presenting cells and MHC cross presentation [140,141]. This efficient trafficking of VLPs and their interaction with the host immune cells induces both innate and adaptive humoral and cellular immune responses, making them particularly attractive vaccine candidates [142,143]. Furthermore, such vaccines present no risk of reversion to virulence nor of dsRNA segment re-assortment with wild virus or live vaccine strains, because they do not contain viral RNA or any of the non-structural proteins. The absence of these viral components also makes it possible to distinguish between vaccinated and infected animals using molecular diagnostic techniques, meaning that VLP vaccines would be DIVA compliant.

The production of both BTV and AHSV VLPs is based on the hypothesis that co-synthesis of proteins VP2, VP3, VP5 and VP7 will result in the spontaneous self-assembly of VLPs via various hydrophobic, electrostatic and covalent interactions [144]. The successful formation and protective efficacy of BTV VLPs produced by the recombinant baculovirus-mediated co-expression of these proteins in insect cells, has been demonstrated [145,146,147]. In the past, most recombinant proteins have been produced either in insect cell lines or in microbial fermentation systems, mammalian cell cultures or transgenic animals. More recently, however, there has been an increased interest in utilizing plant-based expression systems—the so-called “biopharming” or “molecular farming” approach—whereby plants are harnessed as mini-factories to produce useful pharmaceutical proteins [148,149,150,151,152,153]. The high-level plant-based expression of fully-assembled VLPs of BTV-8 has recently been reported [154,155] and the plant-produced VLPs were shown to elicit a strong antibody response in sheep, providing protective immunity against challenge with a BTV-8 field isolate.

An initial investigation regarding the production of AHSV CLPs by co-infection of insect cells with recombinant baculoviruses expressing either AHSV VP3 or VP7 was unsuccessful [156]. CLPs represent the inner and middle protein layers of the virus and lack the outer capsid protein layer composed of proteins VP5 and VP2 which are required for complete VLP formation. In a later study co-expression of AHSV viral proteins and assembly of both AHSV CLPs and VLPs was achieved by co-infection with baculovirus recombinants simultaneously expressing two AHSV capsid proteins, i.e., VP2 and VP3 or VP5 and VP7 [157]. However, the overall VLP yield was very low and precluded quantification.

**Figure 4:**
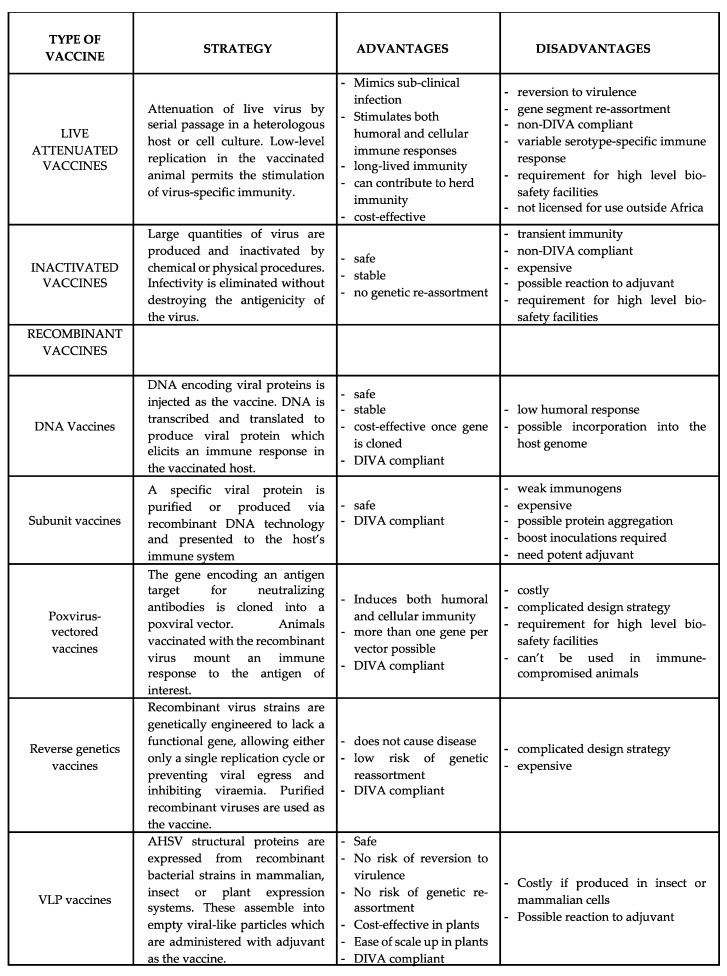
A summary of the various vaccine strategies against African horse sickness.

Recently, the development of a plant-produced AHSV VLP vaccine was described [158,159]. Transient co-expression of the four capsid proteins of two different AHSV serotypes in the common tobacco plant, *N. benthamiana*, resulted in the efficient self-assembly of well-formed AHSV VLPs which were shown to be both safe and highly immunogenic in horses. Confidence in this novel AHSV VLP vaccine hinges largely on the fact that it is a vaccine comprised entirely of protein, free from infectious genetic material, and produced using a cost-effective plant expression system. Furthermore, the vaccine platform mimics field exposure to the naturally immunogenic AHS virion, but without endangering the immunised animal in any way and being completely free of the possibilities of reversion to virulence, or reassortment with other vaccine or wild-type viruses. Sera from horses immunized with AHSV 5 VLPs also elicited a comparative immune response towards AHSV 8, confirming reports of cross protection between these two serotypes and demonstrating the importance of pursuing further investigation into the potential and suitability of this candidate AHSV vaccine. The various vaccine strategies against African horse sickness, together with the advantages and disadvantages of each, are summarized in Figure 4.

## 6. Conclusions

African horse sickness is a lethal and debilitating disease of domestic equids. There is little doubt that the live attenuated vaccine that has been used in South Africa to protect horses against AHSV for the past six decades [160,161] has ensured their continued existence. However, the manufacture of this vaccine uses very old technology and production volumes that cannot meet the current demand. Furthermore, the fact that many horses still contract the disease and often die in spite of vaccination, as well as the fact that this vaccine is not licensed for use outside of the African sub-continent, has led to an increasing demand for a new, safer and more cost-effective vaccine which would not only address the concerns of South African horse owners, but also meet approval in the wider international context where live vaccines for the disease would not be acceptable. Biotechnological advances over the past few decades have paved the way for new generation vaccines which lack the associated negative features of the LAV, and which could potentially serve as adequate replacement vaccines.

An ideal AHSV vaccine would activate both humoral and cell-mediated immune responses and provide rapid and long-lasting protective immunity against all nine serotypes of the virus. It would block viraemia, disallow transmission by the midge vector, ensure that no risk of reversion to virulence nor re-assortment with outbreak strains was possible, and permit accurate differentiation between vaccinated and infected animals. It should be possible to safely, consistently and economically produce sufficient doses of such a vaccine to meet the demands of both the private and rural sectors. Importantly, the vaccine should hold sufficient interest and market potential to capture the attention of the manufacturing industry.

Five types of alternative AHS vaccine platforms have been described in recent years. Two of these, the ECRA (Entry Competent Replication Abortive) [131] and DISA (Disabled Infectious Single Animal) [132] candidate vaccines, stem from research in the area of reverse genetics technology, while the third and fourth are based on modified pox viruses [117,122]. Although the results obtained in experimental trials with these vaccines look promising, issues of cost and scalability have thus far prevented any from being commercialized. The newest VLP candidates have the potential to be as efficacious as the currently used live attenuated vaccine, but without the latter’s accompanying risks and shortcomings. Furthermore, they have the associated benefits of cheap and scalable production processes. However, challenge trials with live virus and further investigation into the development of VLPs of the other AHSV serotypes are required to conclusively demonstrate the protective efficacy of these plant-produced vaccines.

## Patents

The authors AM and ER have filed a patent application protecting the production of chimaeric orbivirus virus-like particles in plants (PCT/IB2017/052236).
